# Cancer Doesn’t Know the Day of the Week: Temporal Trends in Day of Death

**DOI:** 10.21203/rs.3.rs-4708726/v1

**Published:** 2024-07-10

**Authors:** Kanan Shah, Anna Tao, Junzo Chino, Fumiko Chino

**Affiliations:** NYU Langone Health; Tufts University Medical Center; Duke Cancer Center; Memorial Sloan Kettering Cancer Center

**Keywords:** comfort & palliative care, end of life care, symptom management, psychosocial factors

## Abstract

Studies support the existence of psychosomatic phenomena that enable critically ill patients to postpone death until a specific event. We assessed for this effect in cancer by examining variability in deaths at the month and weekend levels using the National Center for Health Statistics database. We found that deaths from cancer were not uniformly distributed temporally. There was a relative 3.3% difference death rate between the peak on Saturday and nadir on Monday, and relative 10.2% difference in rate of death between the peak of deaths in January and nadir in February. The “weekend effect” could be present in 1 in 200 cancer deaths and the “holiday effect” in 1 in 100 cancer deaths. Temporal variation may reflect a small portion of patients are able to “hold on” for a limited amount of time. This uneven distribution of cancer deaths highlights the importance of improving communication and facilitating end-of-life discussions.

## Introduction

Improving end-of-life care remains an opportunity to reduce patient and caregiver trauma during the dying process.(1) Caregivers of patients who die in hospital or ICU settings have higher risk for prolonged grief and posttraumatic stress disorders.(2) Early hospice enrollment (3) and non-hospital death are less physically and emotionally distressing for patients (4), and may enable them to be with family and loved ones before dying.(5)

Studies support the existence of psychosomatic phenomena that enable the critically ill to prolong survival until the arrival of family on weekends or the passage of a major holiday. In the “holiday effect,” dying patients enter a “bargaining phase” to postpone death until a specific event. Studies in Jewish populations show a decrease in expected deaths in the week before Passover, followed by an equal rise afterwards.(6) A similar pattern has been seen around the Harvest Moon Festival amongst Chinese Americans.(7)

Little is known about the existence of this phenomena in patients with cancer. Using a comprehensive national cohort, we examined the variability in deaths at the month and weekend levels for patients with terminal cancer.

## Methods

All deaths due to malignant neoplasms (ICD codes C00-C97) from 2000–2017 were examined using nationally comprehensive deidentified mortality data from the National Center for Health Statistics. Outcomes were days of the week and months of year that death occurred with death on Friday/Saturday/Sunday defined as “weekend death” and death in December/January defined as “holiday death”. Chi-squared tests determined differences by day or month. Multivariate logistic regression examined associations between day or month of death and age, education, race/ethnicity, location of death, marital status, race, and sex. Statistical analyses were performed using SAS statistical software, version 9.4 (SAS Institute Inc).

## Results

**Figure 1:**
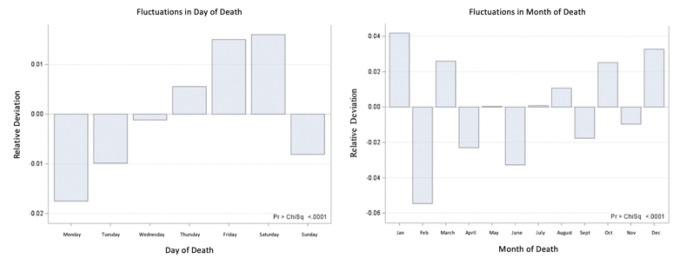
Fluctuations in Cancer Death across Day and Month of Death

A total of 10,305,990 deaths due to cancer in 2000–2017 were recorded. Deaths were not uniform across day of week or month of year (p < 0.001 each) (Fig. 1). Every year, rate of death consistently increased from Monday to Thursday, peaked on Friday and Saturday, and declined on Sunday. There was a relative 3.3% difference in death rate between the peak of deaths on Saturday and nadir on Monday. Additionally, a consistent increase in deaths was seen in December and January, followed by a clear nadir in February with a relative difference of 10.2%. See **Supplemental Table 1** for full details.

Non-hospital deaths including home, nursing facility, and hospice, were more likely to occur on the weekend (adjusted Odds Ratio, aOR from 1.03–1.05, p ≤ 0.01 for all). Hispanic patients (aOR 1.01, p = 0.001) were more likely to die on weekends. Females (aOR 0.99, p = 0.02), those who were divorced (aOR 0.99, p = 0.04), and those with pancreatic cancer (aOR 0.99, p = 0.01), were less likely to die on weekends. Non-hospital death was less likely to occur during Holiday months (aOR from 0.95–0.96, p ≤ for all). Those over 75 years old were more likely to die during Holiday months compared to younger age groups (OR 1.02–1.03, p < 0.05 for both). Cancer type was associated with holiday mortality; those with breast or prostate cancer were more likely to die during the holidays (OR 1.01, p < 0.05 for both), compared to those with lung cancer. Full analysis shown in Table 1.

## Discussion

There is a human element to death. The uneven distribution of cancer deaths in days of the week and months of year suggests a non-biological variation to some deaths as cancer mortality itself should theoretically be date agnostic. These differences may reflect a portion of patients who are able to “hold on” for a limited period to satisfy a personally meaningful moment. Given these relative differences, the “weekend effect” could be seen in up to 1 in 200 cancer deaths and the “holiday effect” in 1 in 100 cancer deaths.

Current literature report conflicting evidence on the ability of patients with cancer to postpone death. One study of cancer deaths in Ohio between 1989–2000 reported no evidence that patients were able to postpone death to survive significant religious (Christmas), social (Thanksgiving), or personal events (individual birthdays).(8) Another examination of cancer deaths in Germany from 1995–2009 supported the existence of the “holiday effect” around Christmas.(9) In this population-level study, we examined all cancer deaths in the US with a focus on the 21st century to provide an updated, comprehensive understanding of cancer death in America. Our results highlight an opportunity to improve end of life care and focus on what matters to patients. If patients can hold on for special moments with family and loved ones, maximizing this quality time is the priority. This is particularly important in the modern era as hospice use is at a historic high, but overall care intensity outside of hospice has increased and the length of hospice stay is low.(10)

Temporal trends were more likely to occur in certain patient populations. Hispanic patients, for example, may have more robust extended family support than other ethnicities (11), a potential incentive for patients to make it to the weekend to see family. Elderly patients may experience a stronger “holiday effect” if given a longer disease trajectory that may allow them to arrange plans for their last days.

Our study is comprehensive by including all deaths from cancer in the US over two decades in the modern era of cancer therapy. Complex interpretations are limited by the absence of documented situations occurring immediately before death, such as treatments received, or end-of-life conversations conducted. The demonstrated temporal trends may also have alternative explanations including seasonal sickness or increased risk of death later in the week due to hospital causes.

## Conclusion

The temporal trends in cancer deaths demonstrated in this study highlight the importance of improving communication and facilitating end-of-life discussions with patients and family members. Adoption of strategies that increase acceptance and lead to early hospice referral may ensure that loved ones have adequate notice to anticipate imminent death (12) and may give patients an improved sense of control during end-of-life transitions.(13) Expedited, safe discharge of the terminally ill presents a chance for patients to settle comfortably into home hospice or a hospice facility and offers a more comfortable, dignified place to die.

## Data Availability

Data derived from a source in the public domain. The data underlying this article are available in: https://www.cdc.gov/nchs/data_access/vitalstatsonline.htm The datasets were derived from sources in the public domain: https://www.cdc.gov/nchs/data_access/VitalStatsOnline.htm#Mortality_Multiple
